# Predictors of Postprandial Hyperglycemia in Non-Diabetic Adult Hospital Visitors: A Cross-Sectional Study Across Religious Groups in Northern Israel

**DOI:** 10.3390/jcm13247866

**Published:** 2024-12-23

**Authors:** Amir Bashkin, Osnat Sharon, Anita Zur, Afif Nakhleh

**Affiliations:** 1 Endocrinology and Diabetes Unit, Galilee Medical Center, Nahariya 2210001, Israel; amirb@gmc.gov.il (A.B.);; 2 The Azrieli Faculty of Medicine, Bar-Ilan University, Safed 1311502, Israel

**Keywords:** diabetes, hospital visitors, postprandial hyperglycemia, screening program

## Abstract

**Background/Objectives:** Ethnocultural differences between Jewish and Arab communities in Northern Israel may contribute to disparities in type 2 diabetes prevalence. Widespread screening strategies, including hospital-based initiatives, are crucial for early detection of hyperglycemia. This study aimed to determine the prevalence of postprandial hyperglycemia and identify its associated factors in a diverse population of non-diabetic adults visiting the Galilee Medical Center, a tertiary care hospital in Northern Israel. **Methods:** Participants were recruited between November 2017 and July 2023 through a voluntary screening program for non-diabetic adult visitors to the hospital. Capillary blood glucose measurements were obtained 1–4 h after a meal using a standardized glucometer. Postprandial hyperglycemia was defined as a blood glucose level ≥147 mg/dL, while postprandial normoglycemia was defined as ≤133 mg/dL. Individuals with glucose levels between 134–146 mg/dL were excluded from the analysis. Additional exclusion criteria included known diabetes, acute illness, corticosteroid use, and pregnancy. Demographic data, lifestyle factors, and health status were recorded. Propensity score matching was employed to ensure comparability between religious groups based on age, gender, and body mass index. Logistic regression analyses were conducted to identify independent predictors of postprandial hyperglycemia. **Results:** 3457 adult visitors underwent postprandial glucose testing and met eligibility criteria. Following propensity score matching, 1845 participants (615 each from Druze, Jewish, and Muslim religious groups) were included in the final analysis. The prevalence of postprandial hyperglycemia was 9.4% in Druze, 6.0% in Jews, and 8.0% in Muslims (*p* = 0.08). Age >50 years was significantly associated with postprandial hyperglycemia in all groups. Obesity was associated with postprandial hyperglycemia in Muslims, with a similar non-significant trend in the Jewish cohort. Self-reported poor health was also associated with postprandial hyperglycemia in Muslims. In the Druze cohort, a low daily intake of daily fresh vegetable consumption was significantly associated with postprandial hyperglycemia. **Conclusions:** This study highlights the feasibility of hospital-based screening for postprandial hyperglycemia among adult visitors and reveals ethnic variations in prevalence and associated risk factors.

## 1. Introduction

Diabetes poses a significant public health challenge, impacting 13% of US adults, with an additional 34.5% having prediabetes. Early detection is critical to minimize complications, yet 8.5 million Americans remain undiagnosed [1]. Notably, the global prevalence of impaired glucose tolerance is estimated at 9.1% (464 million), underscoring the need for heightened awareness and proactive screening measures to prevent diabetes [2].

In 2022, the American Diabetes Association (ADA) lowered the recommended screening age to 35. The ADA also advises screening overweight or obese adults with risk factors [3]. Racial, ethnic, and socioeconomic disparities in type 2 diabetes persist and demand targeted interventions to reduce the burden of this disease [4].

Northern Israel is a region of rich demographic diversity, characterized by the coexistence of Jewish and Arab communities, each with unique cultural and historical practices. The Arab population is predominantly Muslim, with Christian and Druze minorities, often residing in smaller villages, while the Jewish population, primarily of Ashkenazi and Mizrahi origins, is largely urban [5]. Notably, Arab communities exhibit a high prevalence of consanguineous marriage, contributing to endogamy within these groups. These distinct historical and cultural practices have collectively shaped the genetic landscape and diversity observed in Israel [5]. Significant disparities in the prevalence of non-communicable diseases exist among the various population subgroups in Israel [6]. Notably, type 2 diabetes is more prevalent among Israeli Arabs (12%) compared to Israeli Jews (6.2%), with potential underdiagnosis also being more common in the Arab population [7,8].

The ADA consensus panel states that postprandial glucose levels in nondiabetic individuals typically remain below 140 mg/dL and generally return to baseline within 2–3 h following a meal [9]. Postprandial hyperglycemia is characterized by an excessive rise in blood glucose levels following a meal. It is an early indicator of impaired glucose regulation, often preceding fasting hyperglycemia, and is significantly elevated in diabetic patients with high fasting glucose [10]. Several factors contribute to postprandial hyperglycemia including impaired insulin secretion, insulin resistance, increased hepatic glucose production, and incretin dysfunction [10].

Postprandial hyperglycemia prevalence varies across populations due to genetic factors, diet, and lifestyle. People with high carbohydrate intake or genetic susceptibility to impaired insulin secretion may have higher postprandial hyperglycemia prevalence [11]. Several lifestyle and dietary habits play a crucial role. A high consumption of sugar-sweetened beverages leads to rapid glucose absorption and large postprandial glucose excursions [12]. Conversely, an adequate daily consumption of fresh vegetables is generally associated with improved glucose metabolism and may mitigate postprandial glucose spikes [13]. Smoking is linked to increased insulin resistance and impaired glucose metabolism, thereby contributing to elevated postprandial glucose levels [14]. In addition, established comorbidities like hypertension dyslipidemia and obesity are frequently observed in conjunction with postprandial hyperglycemia, underscoring the interconnected pathophysiology of metabolic and cardiovascular disorders [10,15].

Sociocultural factors and health perceptions may indirectly impact postprandial hyperglycemia by shaping health behaviors and resource access. Religiosity, potentially associated with improved glycemic control, influences dietary practices, stress management, and social support [16]. Self-perceived good health correlates with healthier behaviors like regular exercise and balanced diets, contributing to better glucose control [17]. This study aimed to determine the prevalence of postprandial hyperglycemia and identify associated readily measurable factors within a diverse population of adult visitors without a prior diagnosis of diabetes, at the Galilee Medical Center, a tertiary care hospital in Nahariya in Northern Israel.

## 2. Materials and Methods

Data were collected from a voluntary screening program for the early detection of diabetes among adult (≥18 years of age) visitors to the Galilee Medical Center who were not current patients of the facility.

### 2.1. Study Subjects and Definitions

This cross-sectional analysis included adults who visited the Galilee Medical Center within 1–4 h of their most recent meal. Capillary blood glucose levels were assessed using a standardized institutional glucometer (Accu-Chek, Roche, Basel, Switzerland).

In our study, postprandial hyperglycemia was defined as a blood glucose level of ≥147 mg/dL, while postprandial normoglycemia was defined as a blood glucose level of ≤133 mg/dL. These thresholds account for the glucometer’s potential measurement deviation of 10%. These thresholds were chosen to ensure a clear distinction between normoglycemic and hyperglycemic states. Subjects were included in this analysis only if their postprandial blood glucose level was ≤133 mg/dL or ≥147 mg/dL. Individuals with known diabetes, acute illness, corticosteroid use, or pregnancy were excluded. Individuals with postprandial hyperglycemia were referred to their primary care physician for further evaluation and treatment. Data on participants age, gender, weight, height, body mass index (BMI), religion, religiosity, antihypertensive medication use, smoking status, self-reported general health status, and daily consumption of sweetened beverages and fresh vegetables (at least one serving per day) were collected through verbal interviews.

**Figure 1:**
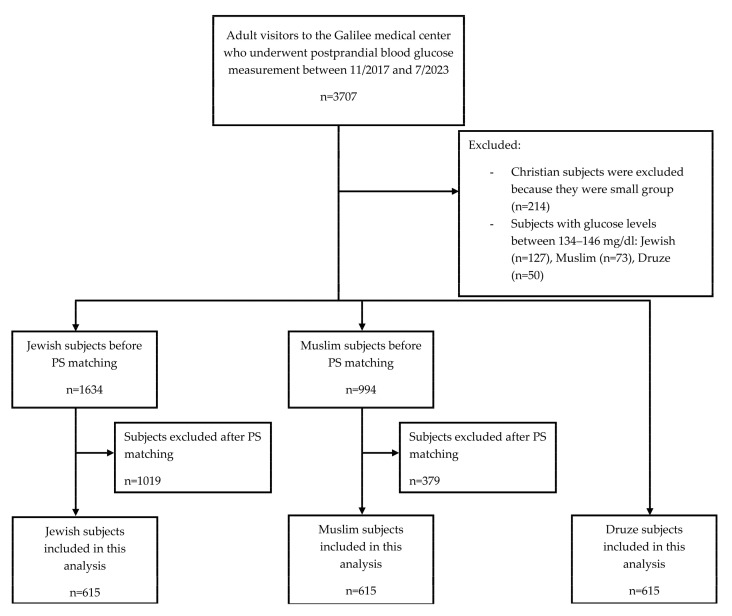
Flow diagram of subjects included in the study. Abbreviations: PS, propensity score.

Between November 2017 and July 2023, 3707 adults underwent postprandial glucose testing at the Galilee Medical Center. Of those, 3457 met the eligibility criteria. This population consisted of 1634 Jewish, 994 Muslim, 615 Druze, and 214 Christian individuals. Due to their smaller sample size, Christians were excluded from this analysis (Figure 1).

The institutional review board and ethics committee granted ethical approval. Due to the program’s screening nature, written informed consent was not required. However, participants received a clear verbal explanation of the screening program and the associated study and were informed of their right to decline or withdraw at any time.

### 2.2. Statistical Analysis

Descriptive data were reported as means ± standard deviations (SD) unless otherwise stated. Propensity score matching was used to match each Druze subject with one Jewish and one Muslim subject based on age, gender, and BMI. The chi-square test was used to compare categorical variables between the religious groups. An independent samples t-test was conducted to compare the continuous variable between groups prior to propensity score matching. The Kruskal–Wallis test was used to compare continuous variables among religious groups after propensity score matching. Multiple regression with backward elimination was used to identify factors most associated with postprandial hyperglycemia within each religious group. The model included nine parameters: age (>50 vs. <50 years), gender (female vs. male), BMI (>30 vs. <30 kg/m^2^), self-identified religiosity (yes vs. no), antihypertensive treatment (yes vs. no), perceived general health (poor vs. good), active smoking (yes vs. no), the daily consumption of sweetened beverages (yes vs. no), and the daily consumption of fresh vegetables (no vs. yes). All statistical analyses were performed using SPSS 25.0 (IBM SPSS version 25, Chicago, Illinois, USA).

## 3. Results

The baseline characteristics of the 3243 participants from the three religious groups (Druze, Jewish, and Muslim) are presented in Table 1.

Propensity score matching was conducted on the Druze, Jewish, and Muslim groups. Covariate balance after propensity score matching was assessed using standardized differences (Table S1).

After matching 615 individuals from each group based on age, gender, and BMI, the final cohort included 63.9% men, with a mean age of 43.5 ± 14 years and a BMI of 27.2 ± 4.7 kg/m^2^. Postprandial hyperglycemia was observed in 7.8% of the cohort. The prevalence of postprandial hyperglycemia was 9.4% for Druze, 6.0% for Jews, and 8.0% for Muslims (*p* = 0.08). Muslim participants reported higher religiosity and had higher smoking rates compared to the other groups, while Jewish participants consumed the fewest daily sweetened beverages. The Druze group had the highest daily intake of fresh vegetables (Table 2).

Table 3 summarizes the results of the univariate logistic regression model used to identify readily measurable factors associated with postprandial hyperglycemia within each group individually. After backward elimination analysis (Table 4), age >50 years was associated with postprandial hyperglycemia across all groups (OR = 2.68, 95% CI [1.49–4.81], *p* = 0.001 for Druze; OR = 2.79, 95% CI [1.35–5.76], *p* = 0.006 for Jews; OR = 3.67, 95% CI [1.90–7.06], *p* < 0.001 for Muslims). Among Muslims, obesity (OR = 2.07, 95% CI [1.10–3.89], *p* = 0.02) was associated with postprandial hyperglycemia. While a similar trend was noted in the Jewish cohort, it did not reach statistical significance (*p* = 0.08). Among Muslims, self-reported poor health (OR = 2.19, 95% CI [1.03–4.66], *p* = 0.04) was also associated with postprandial hyperglycemia. Additionally, for the Druze group, low daily intake of daily fresh vegetable consumption was significantly associated with postprandial hyperglycemia (OR = 2.32, 95% CI [1.16–4.66], *p* = 0.02).

## 4. Discussion

The present study reveals that the hospital setting can serve as a valuable screening point for postprandial hyperglycemia among adult visitors who are not current patients. We examined the prevalence of postprandial hyperglycemia and its associated predictive factors among non-diabetic adult visitors to a tertiary care hospital in Northern Israel. We observed a 7.8% prevalence of postprandial hyperglycemia in the study population.

Measuring postprandial glucose is a valuable tool for the early detection of prediabetes and diabetes, as postprandial hyperglycemia often precedes fasting hyperglycemia and is associated with vascular complications, even in prediabetes [18]. Our study highlights the hospital setting as an opportunity to identify individuals with postprandial hyperglycemia. Following matching for age, gender, and BMI, a statistically non-significant trend towards a higher prevalence of postprandial hyperglycemia was observed in Druze and Muslim participants compared to Jewish participants. This observation aligns with previous studies demonstrating a higher prevalence of diabetes among Arabs compared to Jews in Israel [19,20]. The reasons behind this disparity are likely multifaceted. Dietary habits, such as higher fat and refined carbohydrate consumption among Arabs, may play a role [19]. Additionally, lifestyle factors like lower physical activity levels and less emphasis on preventive health measures could contribute to increased diabetes risk [19]. The underdiagnosis of diabetes, which has been reported to be more prevalent among Arabs than Jews, could also contribute to the observed differences [8]. Genetic predisposition may also be a factor, although further investigation is needed to determine its extent [20].

Across all groups, age emerged as the strongest predictor of postprandial hyperglycemia, consistent with the well-established association between advancing age and increased risk of prediabetes and diabetes [3,21]. The study found no significant association between gender and postprandial hyperglycemia, consistent with the existing literature [22].

Obesity was positively correlated with postprandial hyperglycemia in the Muslim cohort. A similar trend was observed in the Jewish cohort, although it did not reach statistical significance. These findings align with established research indicating a strong association between obesity and the development of prediabetes and diabetes [3,23]. In the Druze cohort, obesity and postprandial hyperglycemia showed no correlation, possibly due to genetic and lifestyle factors. However, further research is needed to confirm this observation.

Self-reported poor health was associated with postprandial hyperglycemia among Muslims. A similar trend was observed in the Druze cohort, but it was not statistically significant. No such association was found in the Jewish cohort. These findings suggest that the relationship between self-reported health and postprandial hyperglycemia may vary across different ethnic and cultural groups.

Interestingly, our study found that a lack of daily fresh vegetable consumption was significantly associated with hyperglycemia exclusively in the Druze cohort. This finding is consistent with existing literature indicating that consuming vegetables before carbohydrates significantly reduces postprandial glucose excursions in individuals with and without type 2 diabetes [13]. However, this association was not observed in the other study groups. This observation warrants further investigation to elucidate the underlying mechanisms and potential dietary, metabolic, and genetic factors contributing to this differential response.

Contrary to expectations, the daily consumption of sweetened beverages was not significantly associated with postprandial hyperglycemia. This unexpected finding could be attributed to the inherent limitations of self-reported dietary data, as participants may not accurately recall or report their consumption habits [24]. Moreover, other dietary factors, such as overall carbohydrate intake and fiber consumption, could modulate postprandial glucose levels, potentially masking the effect of sweetened beverages [25].

The study found no significant association between postprandial hyperglycemia and either antihypertensive medication use or active smoking. While hypertension and smoking have been independently linked to glucose metabolism [14,15], their association with postprandial hyperglycemia in our study might be masked or modified by various factors, including limitations of self-reported data and the influence of other dietary and lifestyle factors.

Our study found no significant association between religiosity and postprandial hyperglycemia. However, previous studies have suggested a potential link between religiosity and improved glycemic control possibly through its influence on lifestyle choices, social support, and stress management [16].

Our study has limitations. The cross-sectional design precludes the establishment of causal relationships between the identified factors and postprandial hyperglycemia. The reliance on self-reported data introduces the possibility of reporting bias. Potential selection bias due to the hospital setting and unmeasured confounders could also influence our results. Several potential confounders like socioeconomic status, more objective measures of diet, physical activity levels, family history of diabetes, comorbidities, and other medications associated with hyperglycemia were not considered. Both travel distance and physical activity after eating may influence postprandial glucose levels and could be confounding factors in this study. Furthermore, postprandial glucose levels can be influenced by various factors including meal composition and individual variability [26]. Finally, our study focused on specific religious groups in a specific region, limiting the generalizability of our findings to other populations. Future research should aim to overcome these limitations by using more objective measures.

## 5. Conclusions

This study suggests that screening for postprandial hyperglycemia in adult hospital visitors can help identify individuals at risk of undiagnosed prediabetes or diabetes, though further testing is needed to confirm a diagnosis. Early detection through such screening initiatives may facilitate timely diagnosis and appropriate management. Additionally, our data indicate ethnic variations in both the prevalence of postprandial hyperglycemia and its associated risk factors. These disparities underscore the need for further investigation to elucidate the specific pathophysiological characteristics and risk profiles associated with postprandial hyperglycemia within distinct ethnic populations. This understanding will facilitate precision medicine approaches that personalize screening, prevention, and treatment based on individual factors, including ethnicity, genetics, and lifestyle.

## Figures and Tables

**Table 1:** Characteristics of participants by religious group before propensity score matching. Abbreviations: BMI, body mass index. Statistical significance is also indicated by asterisks as follows: * *p* < 0.05, ** *p* < 0.01, *** *p* < 0.001.

	Druze (N = 615)	Jews (N = 1634)	Muslims (N = 994)	*p*-Value (Druze vs. Jews)	*p*-Value (Druze vs. Muslims)
Male N (%)	393 (63.9)	763 (46.7)	575 (57.8)	<0.001 ***	0.02 *
Age, Y Mean (SD)	43.5 (13.9)	52.1 (15.8)	41.7 (14.3)	<0.001 ***	0.01 *
BMI, kg/m^2^ Mean (SD)	27.2 (4.5)	26.7 (4.7)	27.7 (5.0)	0.02 *	0.06
Glucose, mg/dL Mean (SD)	111.5 (21.6)	110.0 (23.9)	110.7 (23.2)	<0.001 ***	0.002 **
Postprandial hyperglycemia N (%)	58 (9.4)	96 (5.9)	70 (7.0)	0.004 **	0.09
Glucose, mg/dL Mean (SD)	172 (38)	175 (32)	169 (25)	0.60	0.59
Self-identified as religious N (%)	246 (40.3)	775 (47.9)	604 (61.4)	0.001 **	<0.001 ***
Antihypertensive treatment N (%)	92 (15.3)	318 (20.1)	122 (12.7)	0.01 *	0.15
Tobacco smoking N (%)	180 (29.6)	425 (26.3)	447 (45.2)	0.12	<0.001 ***
Daily fresh vegetable consumers N (%)	525 (85.5)	1304 (80.2)	781 (79.4)	0.005 **	0.002 **
Daily sweetened beverage consumers N (%)	279 (45.7)	510 (31.4)	528 (53.4)	<0.001 ***	0.003 **
Participants perceiving their general health as good N (%)	532 (86.8)	1398 (86.2)	860 (87.1)	0.73	0.88

**Table 2:** Characteristics of participants by religious group after propensity score matching. Abbreviations: BMI, body mass index. Statistical significance is also indicated by asterisks as follows: *** *p* < 0.001.

	Druze (N = 615)	Jews (N = 615)	Muslims (N = 615)	*p*-Value
Male N (%)	393 (63.9)	393 (63.9)	393 (63.9)	1
Age, Y Mean (SD)	43.5 (13.9)	43.7 (14.2)	43.2 (13.7)	0.97
BMI, kg/m^2^ Mean (SD)	27.2 (4.5)	27.3 (4.9)	27.2 (4.6)	0.92
Glucose, mg/dL Median (IQR)	108 (96–122)	103 (93–115)	105 (94–120)	<0.001 ***
Postprandial hyperglycemia N (%)	58 (9.4)	37 (6.0)	49 (8.0)	0.08
Self-identified as religious N (%)	246 (40.3)	287 (47.0)	375 (61.8)	<0.001 ***
Antihypertensive treatment N (%)	92 (15.3)	78 (13.0)	83 (13.9)	0.51
Active tobacco smoking N (%)	180 (29.6)	176 (29.0)	292 (47.8)	<0.001 ***
Daily fresh vegetable consumption (≥1 serving/day) N (%)	525 (85.5)	469 (76.5)	477 (78.3)	<0.001 ***
Daily sweetened beverage consumption (≥1 serving/day) N (%)	279 (45.7)	244 (39.9)	330 (53.9)	<0.001 ***
Participants perceiving their general health as good N (%)	532 (86.8)	550 (89.9)	536 (87.9)	0.24

**Table 3:** Initial logistic regression model for the prediction of postprandial hyperglycemia, using all nine variables. Abbreviations: BMI, body mass index. Statistical significance is also indicated by asterisks as follows: * *p* < 0.05, ** *p* < 0.01, *** *p* < 0.001.

	Druze (N = 615)			Jews (N = 615)			Muslims (N = 615)		
Parameter	OR	95% CI	*p* Value	OR	95% CI	*p* Value	OR	95% CI	*p* Value
Age (>50 vs. <50 years)	2.12	1.09–4.14	0.03 *	3.31	1.54–7.13	0.002 **	3.66	1.79–7.50	<0.001 ***
BMI (>30 vs. <30 kg/m^2^)	1.62	0.88–2.98	0.12	2.48	1.16–5.3	0.02 *	2.12	1.12–4.04	0.02 *
Gender (female vs. male)	0.62	0.30–1.28	0.19	0.62	0.27–1.45	0.27	0.88	0.43–1.82	0.73
Antihypertensive treatment (yes vs. no)	1.18	0.56–2.49	0.66	0.41	0.13–1.31	0.13	0.98	0.44–2.21	0.96
Tobacco smoking (yes vs. no)	0.94	0.47–1.91	0.87	1.65	0.76–3.57	0.21	1.85	0.94–3.62	0.07
Religiosity (religious vs. non-religious)	1.06	0.57–1.95	0.86	1.32	0.64–2.70	0.45	0.63	0.33–1.21	0.17
Self-perception of good health (no vs. yes)	1.92	0.93–3.96	0.08	0.51	0.11–2.27	0.37	2.12	0.96–4.69	0.06
Daily consumption of sweetened beverages (yes vs. no)	1.01	0.55–1.84	0.99	1.24	0.60–2.59	0.56	0.81	0.43–1.54	0.53
Daily consumption of fresh vegetables (no vs. yes)	2.27	1.12–4.6	0.02 *	0.58	0.23–1.47	0.25	0.56	0.22–1.43	0.23

**Table 4:** Final model for predicting postprandial hyperglycemia after backward elimination. Abbreviations: BMI, body mass index; NR, not relevant. Statistical significance is also indicated by asterisks as follows: * *p* < 0.05, ** *p* < 0.01, *** *p* < 0.001.

	Druze (N = 615)			Jews (N = 615)			Muslims (N = 615)		
Parameter	OR	95% CI	*p* Value	OR	95% CI	*p* Value	OR	95% CI	*p* Value
Age (>50 vs. <50 years)	2.68	1.49–4.81	0.001 **	2.79	1.35–5.76	0.006 **	3.67	1.9–7.06	<0.001 ***
BMI (>30 vs. <30 kg/m^2^)	NR	NR	NR	1.9	0.93–3.91	0.08	2.07	1.1–3.89	0.02 *
Tobacco smoking (yes vs. no)	NR	NR	NR	NR	NR	NR	1.75	0.93–3.30	0.08
Self-perception of good health (no vs. yes)	1.84	0.92–3.71	0.09	NR	NR	NR	2.19	1.03–4.66	0.04 *
Daily consumption of fresh vegetables (no vs. yes)	2.32	1.16–4.66	0.02 *	NR	NR	NR	NR	NR	NR

## Data Availability

The data presented in this study are available on request from the corresponding author.

## Supplementary Materials

The following supporting information can be downloaded at: https://www.mdpi.com/article/10.3390/jcm13247866/s1, Table S1: Covariate balance after propensity-score matching assessed using standardized differences.
