# Supplementary material for: Predictors of Postprandial Hyperglycemia in Non-Diabetic Adult Hospital Visitors: A Cross-Sectional Study Across Religious Groups in Northern Israel
Source: J Clin Med. 2024 Dec 23;13(24):7866. doi: 10.3390/jcm13247866 (PMC11727719; doi:10.3390/jcm13247866)
Supplement: Supplementary file 1 [file jcm-13-07866-s001.zip › jcm-3340350-supplementary.pdf]

Supplementary Table

Table S1: Covariate balance after propensity-score matching assessed using standardized differences. Abbreviations: BMI, body mass index

	Druze (N = 615)	Jews (N = 615)	Muslims (N = 615)	Standardized difference (Druze vs Jews), %	Standardized difference (Druze vs Muslims), %
Male N (%)	393 (63.9)	393 (63.9)	393 (63.9)	0	0
Age, Y Mean (SD)	43.5 (13.9)	43.7 (14.2)	43.2 (13.7)	1.6	2.3
BMI, kg/m^2 Mean (SD)	27.2 (4.5)	27.3 (4.9)	27.2 (4.6)	4.4	1.9
